# The Impact of Cholecystectomy in Patients with Post-Bariatric Surgery Hypoglycemia

**DOI:** 10.1007/s11695-024-07325-y

**Published:** 2024-06-06

**Authors:** Daniel Sardão, Hugo Santos-Sousa, Bárbara Peleteiro, Fernando Resende, André Costa-Pinho, John Preto, Eduardo Lima-da-Costa, Paula Freitas, CRI-O Group

**Affiliations:** 1 Faculty of Medicine, University of Porto, Alameda Professor Hernâni Monteiro, 4200-319 Porto, Portugal; 2 Integrated Responsibility Center for Obesity (CRI-O), São João Local Health Unit (ULS), Porto, Portugal; 3 i3S – Institute for Research and Innovation in Health, University of Porto, Porto, Portugal; 4 Centro de Epidemiologia Hospitalar, Unidade Local de Saúde São João, Porto, Portugal; 5 Departamento de Ciências da Saúde Pública E Forenses E Educação Médica, Faculdade de Medicina da Universidade Do Porto, Porto, Portugal; 6 EPIUnit-Instituto de Saúde Pública, Universidade Do Porto, Porto, Portugal; 7 Laboratório Para a Investigação Integrativa E Translacional Em Saúde Populacional (ITR), Universidade Do Porto, Porto, Portugal

**Keywords:** Post-bariatric hypoglycemia, Cholecystectomy, Bariatric surgery, Acarbose, Glucose metabolism, Bile acids, Glucagon-like peptide 1

## Abstract

**Background:**

Metabolic surgery is the foremost treatment for obesity and its associated medical conditions. Nonetheless, post-bariatric hypoglycemia (PBH) emerges as a prevalent complication. PBH pathophysiology implicates heightened insulin and glucagon-like peptide 1 (GLP-1) levels, with bile acids (BA) contributing to GLP-1 release. A plausible association exists between cholecystectomy and PBH, which is attributed to alterations in BA metabolism and ensuing hormonal responses. The objective of this retrospective cohort study was to evaluate the impact of cholecystectomy on PBH pharmacological treatment, diagnostic timelines and metabolic parameters.

**Materials and methods:**

Patients diagnosed with PBH after bariatric surgery were evaluated based on their history of cholecystectomy. Demographic, anthropometric and clinical data were collected. Mixed meal tolerance tests (MMTT) results were compiled to assess metabolic responses.

**Results:**

Of the 131 patients with PBH included in the study, 29 had prior cholecystectomy. The time to PBH diagnosis was similar across groups. Patients with prior cholecystectomy required higher doses of acarbose (*p* = 0.046), compared to those without prior cholecystectomy. Additionally, MMTT revealed higher insulin (*t* = 60 min: *p* = 0.010 and *t* = 90 min: *p* = 0.034) and c-peptide levels (*t* = 60 min: *p* = 0.008) and greater glycemic variability in patients with prior cholecystectomy (*p* = 0.049), highlighting the impact of cholecystectomy on glucose metabolism.

**Conclusion:**

Our study offers novel insights into PBH pharmacotherapy, indicating that PBH patients with a history of cholecystectomy require elevated doses of acarbose for symptom control than PBH patients without such surgical history. Furthermore, our findings underscore the pivotal role of hyperinsulinism in PBH aetiology, emphasizing the significance of the BA-GLP-1-insulin axis.

**Graphical Abstract:**

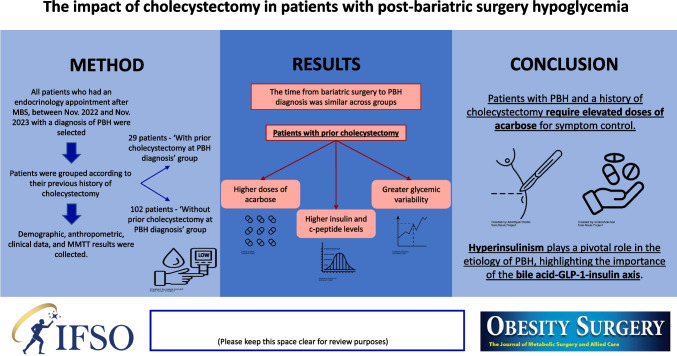

## Introduction

Metabolic and bariatric surgery (MBS), which encompasses a wide range of possible techniques, the main ones being Roux-en-Y gastric bypass (RYGB) and sleeve gastrectomy (SG), has proven to be the most effective and long-lasting treatment for obesity and its associated medical conditions [1]. However, despite the many advantages of this procedure, patients undergoing metabolic surgery, particularly RYGB, can develop some complications, such as post-bariatric surgery hypoglycemia (PBH) and dumping syndrome [2].

The definition of PBH is controversial. Nevertheless, most definitions include a symptomatic hypoglycemic event one to three hours after a meal [3]. This condition has a substantial impact on quality of life and can lead to overeating, thus increasing patients’ weight [4]. More than a third of patients undergoing MBS report symptoms of postprandial hypoglycemia [5]. Nonetheless, severe hypoglycemic episodes occur in less than 12% of patients [6]. PBH is thought to occur several months or even years after metabolic surgery [7].

The physiology of PBH is not yet fully understood, but seems to be explained by the high levels of insulin and glucagon-like peptide 1 (GLP-1) that occur after metabolic surgery. This increase is presumed to be triggered by rapid gastric emptying, leading to an acute glycemic peak. The beta cells of the pancreatic islets are hyperstimulated and consequently release insulin, causing a marked decrease in plasma glucose [8].

The gallbladder plays a crucial role in bile acid (BA) homeostasis. However, cholecystectomy is a procedure performed with high frequency in patients undergoing metabolic surgery, both before and after surgery [9]. One of the consequences of cholecystectomy is an increase in the enterohepatic recycling of BA, increasing their plasma concentration [10]. Following cholecystectomy in patients with history of MBS, postprandial levels of insulin and C4 (a marker of hepatic BA synthesis) are two to three times higher than those of patients with history of MBS who did not undergo cholecystectomy [11]. The metabolism of BA after MBS is not fully known, but it is recognised that they act on cells in the intestinal wall by activating the farnesoid x receptor (FXR) and Takeda G protein-coupled receptor 5 (TGR5), which potentiates the release of GLP-1 [12]. BA also induce an increase in the fibroblast growth factor (FGF19) concentration, which in turn enhances glycogen synthesis [13].

There is a paramount importance to understand the significance of cholecystectomy in patients diagnosed with PBH; however, there is very little scientific evidence on this subject.

The objective of this study was to assess the impact of cholecystectomy in patients with PBH. To do this, we sought to evaluate possible differences in the time interval between MBS and the onset of PBH, in the results of mixed-meal tolerance tests (MMTT) and also the requirement and dose of pharmacologic interventions to control hypoglycemic symptoms in patients with PBH, with or without cholecystectomy at the diagnosis of PBH.

## Materials and Methods

### Patients

All patients who had an endocrinology appointment after MBS at our centre, between November 2022 and November 2023, were screened, and those with a diagnosis of PBH were selected (*N* = 131).

We considered a diagnosis of PBH to be the existence of a history of autonomic and/or neuroglycopenic symptoms at least 6 months after MBS, concomitantly associated with a plasma glucose level < 55 mg/dL 1 to 3 h after a meal. Symptoms subside upon restoration of plasma glucose concentration to physiological levels [3]. Plasma glucose levels during symptomatic episodes were reported by patients (*n* = 32) or objectified during MMTT (*n* = 65) with a standardised liquid meal (Fresubin Energy Drink, 200 ml, 300 kcal [50E% carbohydrate, 15E% protein and 35E% fat]; Fresenius Kabi Deutschland, Bad Homburg, Germany), based on macronutrient composition in accordance with post-bariatric surgery nutritional recommendations [14, 15]. Additionally, hyperinsulinemia (plasma insulin levels > 50 μU/L) during the MMTT was one of the criteria used to support the diagnosis of PBH [16]. On the one hand, headaches, muscle weakness, difficulty concentrating, confusion, convulsions, slurred speech, coma, or altered state of consciousness were all considered to be neuroglycopenic symptoms. On the other hand, sweating, tremors, palpitations, paresthesia and hunger were considered to be autonomic symptoms [16–18]. All patients reported at least one of the above-mentioned symptoms.

Inclusion criteria included a history of previous MBS (RYGB [*n* = 120] or SG [*n* = 11]), stable weight (defined as a change of less than 10% over the last 6 months), HbA1c < 6.5% and fasting plasma glucose < 126 mg/dL at the time of diagnosis of PBH. Exclusion criteria were ongoing pregnancy, taking antidiabetics (except in the case of PBH treatment), diagnosis of diabetes after MBS, history of gastrointestinal surgery (except in the case of MBS and cholecystectomy), chronic kidney disease, or previous diagnosis of any medical condition that could lead to postprandial hypoglycemia.

This was a retrospective cohort study. The study protocol was reviewed and approved by the local Ethics Committee (403–2023).

The following data was acquired from the electronic medical records for all the patients: demographics, anthropometrics, clinical presentation of symptomatic hypoglycemic episodes, history of cholecystectomy, laboratory data, information of MBS and also the time interval between surgery and the onset of hypoglycemia and therapy.

### Study Groups

Patients were selected and grouped according to their previous history of cholecystectomy. Those patients who had undergone cholecystectomy before the diagnosis of PBH were allocated to the ‘With prior cholecystectomy at PBH diagnosis’ group (*n* = 29; a total of 21 patients had prior cholecystectomy to MBS [the median time between cholecystectomy and MBS was 15.9 months] and 8 patients who had undergone cholecystectomy after MBS [the median time between MBS and cholecystectomy was 63.4 months]), and the remaining patients, including those who had undergone cholecystectomy after diagnosis of PBH (*n* = 7), were allocated to the ‘Without prior cholecystectomy at PBH diagnosis’ group (*n* = 102).

### Calculations

The percentage of total weight loss (%TWL) was calculated as [(preoperative weight − weight at PBH diagnosis) ÷ (weight at PBH diagnosis) × 100] and the percentage of excess body mass index (BMI) loss (%EBMI) was determined as [(preoperative BMI − BMI at PBH diagnosis) ÷ (preoperative BMI − 25) × 100], with 25 kg/m^2^ as the target BMI.

Homeostasis model assessment indexes (HOMA2) were determined using the HOMA Calculator version 2.2.3 (http://www.dtu.ox.ac.uk, accessed January 2024), which measures beta cell function (HOMA2-B), peripheral insulin sensitivity (HOMA2-S) and insulin resistance (HOMA2-IR).

The nadir corresponds to the minimum value during the MMTT, and the peak corresponds to the maximum value during the MMTT.

The maximum-to-minimum glucose ratio (MMGR) was calculated to evaluate plasma glucose variation (maximum glycemic value ÷ minimum glycemic value, during the MMTT).

### Statistical Analysis

All analyses were carried out using Stata® IC 15.1 (Stata Corp, College Station, TX, USA).

Continuous data were summarised as being mean ± standard deviation if the variables were normally distributed or median and interquartile ranges if the variables did not follow a normal distribution. Normality of continuous variables was assessed using the Shapiro–Wilk test. In the case of continuous variables that follow a normal distribution, the two groups were compared using Student’s *t*-test and for those who did not follow a normal distribution, we used Kruskal–Wallis test. Categorical data were presented as counts and proportions and were compared using Pearson χ2.

All *p* values < 0.05 were considered to be statistically significant.

## Results

The characteristics of patients with and without prior cholecystectomy at PBH diagnosis are summarised in Table 1. The age at PBH diagnosis and the age at MBS in patients with prior cholecystectomy was slightly higher than those of patients without prior cholecystectomy (*p* = 0.008 and *p* = 0.029, respectively). No statistically significant differences were found in the other demographic or anthropometric parameters. A total of 21 individuals had been diagnosed with type 2 diabetes prior to MBS. The levels indicating beta cell function (HOMA2-B), insulin sensitivity (HOMA2-S) and peripheral insulin resistance (HOMA2-IR) were not significantly different between the two groups (*p* = 0.832, *p* = 0.703 and *p* = 0.741, respectively).

**Table 1:** Patient characteristics, overall and according to prior cholecystectomy at PBH diagnosis

	Total [n = 131]	Without prior cholecystectomy at PBH diagnosis [*n* = 102]	With prior cholecystectomy at PBH diagnosis [*n* = 29]	*p* value
Female, *n* (%)	120 (91.6)	92 (90.2)	28 (96.6)	0.276
Age at MBS, years	40.41 ± 9.57	39.44 ± 9.30	43.83 ± 9.88	**0.029**
RYGB, *n* (%)	120 (91.6)	92 (90.2)	28 (96.6)	0.521
History of T2DM before MBS, *n* (%)	21 (16.0)	16 (15.7)	5 (17.2)	0.840
BMI before MBS (Kg/m^2^)^a^	42.00 (38.65, 45.34)	41.91 (38.57, 45.31)	42.32 (39.45, 46.20)	0.797
BMI at PBH diagnosis (Kg/m^2^)	27.00 (25.02, 30.50)	27.15 (24.77, 30.80)	26.90 (25.85, 29.70)	0.896
Age at PBH diagnosis, years	43.18 ± 9.68	42.00 ± 9.26	47.34 ± 10.14	**0.008**
%EBMIL (%)^a^	83.99 ± 24.65	84.32 ± 24.95	82.81 ± 23.98	0.773
%TWL (%)^a^	53.34 ± 22.47	53.72 ± 21.91	52.00 ± 24.66	0.719
HOMA2-B (%)^b^	97.70 (76.20, 129.70)	97.40 (75.90, 130.00)	98.20 (77.60, 114.60)	0.832
HOMA2-S (%)^b^	128.60 (89.20, 202.30)	128.60 (89.20, 202.30)	127.55 (94.50, 188.30)	0.703
HOMA2-IR^b^	0.78 (0.49, 1.12)	0.78 (0.49, 1.12)	0.79 (0.53, 1.06)	0.741

Data are presented as means ± SD or median (25th percentile, 75th percentile) or number (%). Statistically significant differences (*p* value < 0.05) are highlighted in bold

*PBH*, post-bariatric surgery hypoglycemia; *MBS*, metabolic and bariatric surgery; *RYGB*, Roux-en-Y gastric bypass; *T2DM*, type 2 diabetes mellitus; *BMI*, body mass index; *EBMIL*, excess BMI loss; *TWL*, total weight loss; *HOMA2-B*, homeostasis model assessment for β-cell function; *HOMA2-S*, homeostasis model assessment for insulin sensitivity; *HOMA-IR*, homeostasis model assessment for insulin resistance

^a^BMI before MBS was unknown for one patient in the ‘Without prior cholecystectomy at PBH diagnosis’ group, and therefore it was not possible to calculate %EBMIL and %TWL for this particular patient

^b^HOMA2-B, HOMA2-S and HOMA2-IR were not calculated in 32 patients (*n* = 11 in the ‘With prior cholecystectomy at PBH diagnosis’ group, and *n* = 21 in the ‘Without prior cholecystectomy at PBH diagnosis’ group) who had not undergone a mixed-meal tolerance test (MMTT)

### Metabolic Responses During MMTT

**Fig. 1:**
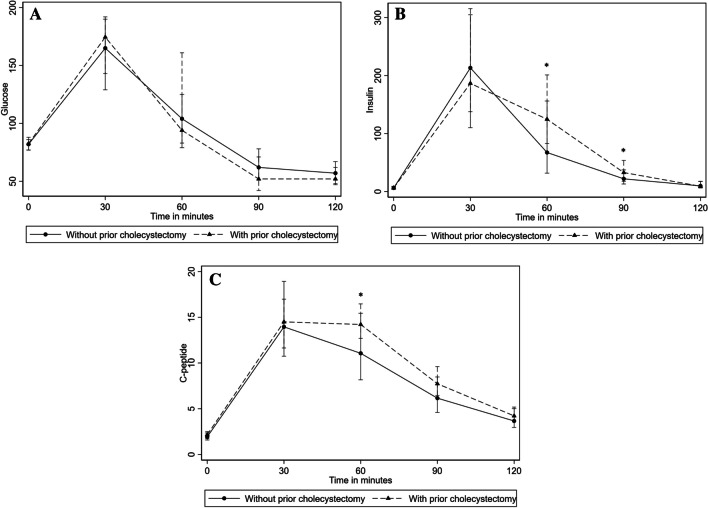
Glucose (**A**), insulin (**B**) and c-peptide peripheral levels (**C**) during MMTT, according to prior cholecystectomy at PBH diagnosis. Data are presented as median (25th percentile, 75th percentile). Statistically significant differences are marked as *p < 0.05

Fasting glucose levels, peak and nadir during the MMTT were similar between the two groups (*p* = 0.692, *p* = 0.400 and *p* = 0.109, respectively, Table 2). No differences were observed at minutes 30, 60, 90 and 120 (Fig. 1A). However, glycemic variability showed statistically significant differences between the two groups, with the MMGR being 24% higher in the group ‘with prior cholecystectomy’ (*p* = 0.049, Table 2).

**Table 2:** Metabolic responses to mixed meal tolerance tests

	Total [*n* = 99]	Without prior cholecystectomy at PBH diagnosis [*n* = 81]	With prior cholecystectomy at PBH diagnosis [*n* = 18]	*p* value
Glucose				
Fasting (mg/dL)	81.90 (76.86, 86.94)	81.90 (76.86, 85.86)	82.44 (76.86, 87.84)	0.692
Peak (mg/dL)	166.68 ± 46.62	164.70 ± 45.90	174.96 ± 49.86	0.400
Nadir (mg/dL)	50.04 (39.96, 52.96)	50.94 (39.96, 54.94)	45.00 (41.94, 50.94)	0.109
MMGR	3.24 (2.55, 4.15)	3.07 (2.47, 4.10)	3.81 (3.44, 4.30)	**0.049**
Insulin				
Fasting (μU/mL)	6.10 (3.90, 8.90)	6.10 (3.90, 8.90)	6.15 (4.40, 8.20)	0.653
Peak (μU/mL)	224.40 (137.70, 308.20)	224.60 (121.60, 307.10)	217.80 (171.00, 315.50)	0.446
C-peptide				
Fasting (ng/mL)	1.98 (1.59, 2.41)	1.93 (1.58, 2.35)	2.16 (1.73, 2.50)	0.291
Peak (ng/mL)	15.46 (12.06, 19.14)	15.39 (11.59, 19.39)	16.12 (14.24, 16.98)	0.272
Nadir (ng/mL)	1.60 (1.28, 2.04)	1.59 (0.99, 1.99)	1.92 (1.55, 2.12)	0.062

Data are presented as means ± SD or median (25th percentile, 75th percentile). Statistically significant differences (*p* value < 0.05) are highlighted in bold

*PBH*, post-bariatric surgery hypoglycemia; *MMGR*, minimum-to-maximum glucose ratio

The ‘with prior cholecystectomy’ group had significantly higher insulin levels (*t* = 60 min: *p* = 0.010 and *t* = 90: *p* = 0.034) (Fig. 1B). However, despite this, there were no differences in fasting and peak insulin levels (*p* = 0.653 and *p* = 0.446, respectively, Table 2).

C-peptide levels showed a similar trend to insulin, with significantly higher levels in the ‘with prior cholecystectomy’ group at minute 60 (*p* = 0.008, Fig. 1C). At minutes 30, 90 and 120, the levels of c-peptide were also higher in the ‘with prior cholecystectomy’ group, although the differences were not statistically significant (*p* = 0.758, *p* = 0.063 and *p* = 0.406, respectively, Fig. 1C). C-peptide parameters, such as fasting levels, peak and nadir, were comparable between the groups (*p* = 0.291, *p* = 0.272 and *p* = 0.062, respectively, Table 2).

### Pharmacological Treatment and Time from MBS to Diagnosis of PBH

The median time to diagnosis of PBH (time interval between MBS and PBH diagnosis) of all patients was 25 months (2 years and 1 month). There were no differences between the groups with and without prior cholecystectomy (*p* = 0.167, Table 3). A total of 62.8% of the patients in the ‘without prior cholecystectomy’ group required acarbose associated with dietary modifications to control hypoglycemic episodes, and in the cases of the ‘with prior cholecystectomy’ group, the percentage of patients who required acarbose was higher (79.3% vs 62.8%, respectively), albeit the difference was not statistically significant (*p* = 0.096, Table 3). The proportion of patients in the ‘with prior cholecystectomy’ group who required medication with a dose greater than or equal to 150 mg/day to control hypoglycemic symptoms was significantly higher than in the ‘without prior cholecystectomy’ group (69.6% vs 45.3%, respectively, *p* = 0.046, Table 3). A sensitivity analysis was conducted and showed no differences related to sex or type 2 diabetes prior to MBS in the need for pharmacological therapy or the requirement for medication with a dose greater than or equal to 150 mg/day. Insufficient weight loss has been previously defined as %TWL < 20 [19, 20]. Thus, setting a cutoff point at 20% of TWL, no significant differences were observed. When the analysis was restricted to patients who had undergone RYGB, significant differences were verified, consistent with the overall results (the data are not shown).

**Table 3:** Time from metabolic and bariatric surgery to diagnosis of post-bariatric hypoglycemia and pharmacological therapy

	Total [*n* = 131]	Without prior cholecystectomy at PBH diagnosis [*n* = 102]	With prior cholecystectomy at PBH diagnosis [*n* = 29]	*p* value
Time interval between MBS and PBH diagnosis, months	25 (15, 41)	24 (14, 40)	36 (17, 46)	0.167
Need of pharmacological therapy—acarbose, *n* (%)^a^	87 (66.4)	64 (62.8)	23 (79.3)	0.096
≥ 150 mg/day (%)^b^	45 (51.7)	29 (45.3)	16 (69.6)	**0.046**

Data are presented as median (25th percentile, 75th percentile) or number (%). Statistically significant differences (*p* value < 0.05) are highlighted in bold

*MBS*, metabolic and bariatric surgery; *PBH*, post-bariatric surgery hypoglycemia

^a^All patients controlled hypoglycemic symptoms either through dietary modifications alone, or in conjunction with the use of acarbose

^b^The dose of acarbose that was considered for grouping patients at ≥ 150 mg/day was the minimum effective dose for controlling hypoglycemic episodes

## Discussion

This study provides support for hypothesis that hormonal alterations caused by cholecystectomy have an impact on the diagnosis and pharmacological treatment of patients with PBH. It is known that BA, through the activation of FXR and TGR5, stimulate the production of GLP-1, an insulinotropic hormone, and hence induce changes in glucose metabolism [12, 18, 21]. Alterations in the kinetics of the BA enterohepatic cycle play a role in the development of PBH [22]. Accordingly, we theorise that the absence of the gallbladder in patients with PBH could alter the kinetics of BA and consequently the respective time from MBS to the diagnosis of PBH and its treatment. One previous study showed that patients with history of MBS and cholecystectomy had higher postprandial levels of insulin and C4 (a marker of hepatic BA synthesis) than patients with history of MBS who did not undergo cholecystectomy [11]. Considering this finding, we grouped patients with PBH according to their previous history of cholecystectomy and assessed the dynamics of glucose, insulin and c-peptide during MMTT. A cut-off value of 55 mg/dL was established to define hypoglycemia, in line with previous studies [14, 23, 24]. Postprandial hypoglycemia depends on the characteristics of the meal [25]; however, despite the use of a standardised meal, there is no assurance it can replicate the symptoms that led to the diagnosis of PBH for each patient. In line with a previous study [11], the results showed higher insulin and c-peptide levels in the ‘with prior cholecystectomy’ group, particularly at minutes 60 and 90. However, there were no differences at fasting, peak, and nadir. In turn, MMGR was significantly higher in the ‘with prior cholecystectomy’ group. In both groups, the highest glucose levels were recorded at minute 30, the lowest being at minute 90. The ‘with prior cholecystectomy’ group had higher glucose values at minute 30, and lower ones at minute 90 than the ‘without prior cholecystectomy’ group, reflecting the significantly higher insulin levels at minutes 60 and 90, which thus explains the larger degree of glycemic variability in this group. No clinical differences between the groups could explain the different responses in the MMTT to the same stimulus. The age at MBS and at diagnosis of PBH in the ‘with prior cholecystectomy’ group was slightly higher. However, there is no evidence that a difference of 5 years in itself causes different responses to the MMTT [26]. Our findings therefore reinforce the role of hyperinsulinism as the central mechanism of PBH and the importance of the BA-GLP-1-insulin axis [27–29]. Indeed, a previous study has shown that blocking the GLP-1 receptor can reduce episodes of PBH [24], while others have described that GLP-1 does not play an essential role in the development of PBH [23, 30]. GLP-1 receptor agonist analogs (GLP-1 RA) have been observed to improve hypoglycemic episodes; however, the entire mechanism remains incompletely understood [30–33]. While endogenous GLP-1 acts as an inhibitor of glucagon secretion during hyperglycemic episodes, some studies indicate that exogenous GLP-1 exerts a stimulatory effect on glucagon secretion during hypoglycemia in healthy individuals [34, 35]. The beneficial effect of GLP-1 on PBH may be biphasic, initially inhibitory and later becoming stimulatory of glucagon secretion during hypoglycemia. Additionally, GLP-1 RA has been shown to reduce small intestine motility and delay intestinal transit, potentially enhancing hypoglycemic episodes [36, 37]. Another conceivable mechanism is that long-term treatment with GLP-1 RA could mitigate the effect of postprandial endogenous GLP-1 [30, 31].

The gallbladder is essential for BA homeostasis. Cholecystectomy has been associated with an increase in postprandial BA synthesis and C4, which is a biomarker of synthesis [38]. Considering the increase in GLP-1 and FGF19, which consequently leads to an increase in insulin induced by BA in patients with history of MBS, our aim was to see whether the time interval until the diagnosis of PBH was shorter in patients who had undergone cholecystectomy. Indeed, to the best of our knowledge, this is the first study to describe the early onset of the diagnosis of PBH in patients with prior cholecystectomy versus those who had not undergone cholecystectomy previously. We found no significant differences in the time to diagnosis; however, the time interval to diagnosis was longer in the cases of the ‘with prior cholecystectomy’ group. The median age at PBH diagnosis of all patients was 43 years, and the median time to diagnosis of PBH was 25 months. This is in line with a previous study that reported a median age at PBH diagnosis of 46 years and a longer median time to diagnosis of PBH, 40.6 months [39]. PBH onset and severity are highly diverse. Based on our findings, these alterations do not seem enough to shorten the time from MBS to the development of PBH, even though cholecystectomy induces hypoglycemic kinetic changes in BA.

Modifying dietary habits is the primary treatment for PBH. The nutritional composition of meals impacts glucose and insulin dynamics, depending on the glycemic index, which influences the glycemic peak and insulin secretion [17, 18, 40–42]. Nutritional requirements for patients after MBS vary according to their height, weight, age, and type of bariatric intervention. A daily macronutrient distribution of 45% carbohydrates, 25% proteins and 30% fats is recommended [43, 44]. Hypoglycemic episodes are more likely to be triggered by meals that are low in protein and high in sugar content [45]. Indeed, according to the Society for Endocrinology guidelines for the diagnosis and management of PBH [46], it is recommended to consume at least 60 g/day of protein, although higher intake may be necessary. When consumed concomitantly with carbohydrates, there is a decrease in gastric emptying and intestinal motility, thereby reducing hypoglycemic episodes. Patients with history of MBS are advised to consume fibre to reduce carbohydrate absorption, limit foods with a high glycemic index, prefer complex carbohydrates, and have a diet fractioned in frequent small meals [44, 47–49]. Additionally, maintaining a food diary that includes records of hypoglycemic episodes and the foods consumed prior to these episodes can be beneficial [47]. Nonetheless, these dietary measures may only provide limited benefits for patients with severe symptoms, necessitating the addition of pharmacological therapy [50].

Pharmacological therapy for PBH consists of acarbose, diazoxide, octreotide, GLP-1 RA and pasireotide [51]. The patients included in this study controlled their hypoglycemic episodes either through dietary modifications alone or in conjunction with the use of acarbose, and none required surgical treatment. Considering the metabolic alterations outlined above resulting from cholecystectomy, we aimed to investigate whether a higher proportion of patients in the ‘with prior cholecystectomy’ group required acarbose than those ‘without prior cholecystectomy’. In addition, we studied whether those patients in the ‘with prior cholecystectomy’ group who were medicated with acarbose required a higher dose to control symptoms. According to the international consensus on diagnosing and managing dumping syndrome [52], the minimum dose of acarbose indicated for maintenance therapy is 150 mg/day. However, in this study, several patients required a dose lower than 150 mg/day to control their symptoms, which aligns with a previous study [53]. Therefore, we found that the proportion of patients in the ‘with prior cholecystectomy’ group who required a dose of 150 mg/day or more was significantly higher than the other group. Acarbose, an α-glucosidase inhibitor, lowers glucose, insulin and GLP-1 levels [54]. It blocks the hydrolysis of polysaccharides, oligosaccharides and disaccharides into monosaccharides, thus delaying and attenuating the glucose and insulin peak [53]. The observed disparity in doses between the two groups aligns with the heightened glycemic variability described during the MMTT in individuals with a history of cholecystectomy. This study was a pioneer in comparing the dose of acarbose required to reduce hypoglycemic episodes in patients with and without prior cholecystectomy at diagnosis of PBH. The overactivation of the BA-GLP-1-insulin axis can partly explain the difference found in the acarbose dose. Therefore, considering the potential association between cholecystectomy and PBH, it may be prudent to initiate treatment with acarbose at higher doses in patients with a history of cholecystectomy.

This study presents some limitations that must be acknowledged. We recognise that the number of patients with history of cholecystectomy was relatively small and therefore future studies should contemplate larger samples, which would be important to validate our findings. This study conducted a retrospective analysis on a cohort from a single medical centre in northern Portugal, and thus caution should be exercised when generalising the findings to other populations. Additionally, the patients in our study had no dietary restrictions in the days before the MMTT, which may influence the research outcome. Due to the retrospective design, bias and confounding may have been introduced, representing a scenario which is less likely in a prospective study design. Considering the retrospective design, certain data points are lacking, as they were originally documented for clinical monitoring purposes rather than for the explicit intent of this study. Nevertheless, this study was carried out in a teaching hospital that is recognised as being a reference centre for bariatric surgery in Portugal. The greatest strength of this study is that it provides insights into the differences in pharmacological treatment and hormonal responses during MMTT in PBH patients with and without prior cholecystectomy at PBH diagnosis.

## Conclusion

Our study provides new insights into the pharmacological treatment of PBH by showing that PBH patients with prior cholecystectomy require higher doses of acarbose to control hypoglycemic symptoms when compared to PBH patients who had not undergone prior cholecystectomy. Additionally, our research reinforces the role of hyperinsulinism as being the main mechanism of PBH and also the importance of the BA-GLP1-insulin axis.

## Data Availability

All the data supporting the findings of this study are available from the authors upon reasonable request.
